# Seroprevalence and risk factors of Q fever in goats on commercial dairy goat farms in the Netherlands, 2009-2010

**DOI:** 10.1186/1746-6148-7-81

**Published:** 2011-12-30

**Authors:** Barbara Schimmer, Saskia Luttikholt, Jeannine LA Hautvast, Elisabeth AM Graat, Piet Vellema, Yvonne THP van Duynhoven

**Affiliations:** 1 Centre for Infectious Disease Control, National Institute for Public Health and the Environment, Bilthoven, the Netherlands; 2 Quantitative Veterinary Epidemiology Group, Wageningen Institute of Animal Sciences, Wageningen University, Wageningen, The Netherlands; 3 Department of Small Ruminant Health, Animal Health Service, Deventer, The Netherlands; 4 Academic Collaborative Centre AMPHI, Department of Primary and Community Care, Radboud University Nijmegen Medical Centre, the Netherlands

**Keywords:** *Coxiella burnetii*, small ruminants, seroprevalence, risk factors, zoonosis, goat

## Abstract

**Background:**

The aim of this study was to estimate the seroprevalence of *Coxiella burnetii *in dairy goat farms in the Netherlands and to identify risk factors for farm and goat seropositivity before mandatory vaccination started. We approached 334 eligible farms with more than 100 goats for serum sampling and a farm questionnaire. Per farm, median 21 goats were sampled. A farm was considered positive when at least one goat tested ELISA positive.

**Results:**

In total, 2,828 goat serum samples from 123 farms were available. Farm prevalence was 43.1% (95%CI: 34.3%-51.8%). Overall goat seroprevalence was 21.4% (95%CI: 19.9%-22.9%) and among the 53 positive farms 46.6% (95%CI: 43.8%-49.3%). Multivariable logistic regression analysis included 96 farms and showed that farm location within 8 kilometres proximity from a bulk milk PCR positive farm, location in a municipality with high cattle density (≥ 100 cattle per square kilometre), controlling nuisance animals through covering airspaces, presence of cats or dogs in the goat stable, straw imported from abroad or unknown origin and a herd size above 800 goats were independent risk factors associated with Q fever on farm level. At animal level almost identical risk factors were found, with use of windbreak curtain and artificial insemination as additional risk factors.

**Conclusion:**

In 2009-2010, the seroprevalence in dairy goats in the Netherlands increased on animal and farm level compared to a previous study in 2008. Risk factors suggest spread from relatively closely located bulk milk-infected small ruminant farms, next to introduction and spread from companion animals, imported straw and use of artificial insemination. In-depth studies investigating the role of artificial insemination and bedding material are needed, while simultaneously general biosecurity measures should be updated, such as avoiding companion animals and vermin entering the stables, next to advice on farm stable constructions on how to prevent introduction and minimize airborne transmission from affected dairy goat farms to prevent further spread to the near environment.

## Background

Q fever is a zoonosis caused by *Coxiella burnetii*, an intracellular Gram-negative bacterium. From spring 2007 until the end of 2009, large community outbreaks of Q fever with over 3500 notified cases occurred in the Dutch population, mainly in the south-eastern provinces of the Netherlands [1,2]. The main transmission route is through inhalation of contaminated aerosols. Climatic conditions play a role as dry and windy conditions are favourable for transmission of the bacterium [3]. *C. burnetii *is very resistant to heat, drought and disinfectants [4]. Domestic ruminants are the primary animal reservoirs for *C. burnetii *for human infections. In addition, outbreaks due to parturient cats and dogs are described [5,6]. When infected animals give birth, large numbers of *C. burnetii *can be shed, but shedding of the bacterium can also occur via urine, faeces and milk, and is different between ruminant species in duration and importance of shedding routes [7]. An infection is usually asymptomatic in cattle, while in dairy goats and dairy sheep an infection may result in abortion or stillbirth [4], often without preceding symptoms. Q fever affected goat herds can show abortion rates up to 90% [8,9].

Dairy goats are considered the predominant source of the community Q fever epidemics in the Netherlands since 2007 [2,10]. The overall goat density in the Netherlands is 38 goats per square kilometre and the total number of goats has increased six-fold from 61.000 in 1990 up to 374.000 in 2009. In the period 2000 until 2009, dairy goat farming has increased almost 3-fold from 98.000 up to 274.000 dairy goats and is especially concentrated in the southern parts of the Netherlands [11]. In the Netherlands, dairy goats are mainly kept year-round in deep litter houses, with partially open walls or roofs. During 2005-2009, Q fever abortion waves were reported on 28 dairy goat farms and 2 dairy sheep farms with abortion percentages varying between 10 and 60% [12]. Human incidence of acute Q fever was highest each spring (April-June), following the main lambing season (December-April) [2]. In order to reduce the risk of exposure from *C. burnetii-*infected small ruminants to humans, mandatory vaccination started in the epicentre of the human outbreak in the southeast of the Netherlands from April 2009 onwards following a voluntary small ruminant vaccination campaign in a more restricted area in the fall of 2008. The 2009 vaccination campaign targeted all dairy goat and dairy sheep farms with at least 50 animals, all open farms (petting zoos, care farms) and all known clinically infected farms since 2005. Studies evaluating the effect of vaccination are promising, especially in nulliparous animals [13]. In October 2009, mandatory bulk milk monitoring using PCR was implemented on all dairy goat and dairy sheep farms with more than 50 animals, to actively detect *C. burnetii-*positive farms, next to the mandatory notification of abortion waves [2]. As of 25 April 2011, 96 dairy goat farms (about 25% of the about 360 large dairy goats farms in 2010) and 2 dairy sheep farms (5%) were found to be bulk milk-positive [14]. In European countries where studies have been done, the seroprevalence in goats in general varies between 6.5% and 48.2%, but is reported up to 75% if sampling is done in shedder goats such as reported in France [15,16]. Farm prevalences were 42.9% in Northern Ireland, 43.0% in a study from Italy and 47.0% in northern Spain [17-19]. Seroprevalences may vary widely within countries as demonstrated in the south-east of France, where at 39 farms without Q fever abortions during the last five years within-herd rates ranged from 0-98% [20]. In 2008, in the Netherlands, the overall Q fever seroprevalence in a convenience sample of 3,134 samples from 442 goat farms submitted for the *Brucella melitensis *monitoring program was 7.8% (95%CI 6.9-8.8%) [12]. The seroprevalence was 11.4% in the southeastern part of the Netherlands compared to 5.3% in the rest of the country. Seroprevalence was higher among the 1,290 dairy goats compared to the 1,844 non-dairy goats (14.7%, 95% CI 2.8%-16.6% versus 3.0%, 95%CI 2.2%-3.8%). The farm prevalence (at least one goat testing positive), was 17.9% (95%CI 14.2-21.5). The average within-herd prevalence on a positive dairy goat farm was 32.1% (95%CI 28.4-35.9%) (van den Brom R, Moll L, Vellema P: Q fever seroprevalence in sheep and goats in the Netherlands in 2008, submitted). Risk factor studies for Q fever in dairy goat farms in the Netherlands have not been done. The aim of this study was to assess the actual magnitude of the spread of *C. burnetii *among small ruminants following the (what turned out to be the peak-) epidemic season in humans in 2009, through testing of sera from a nationwide representative sample of dairy goat farms prior to the start of mandatory vaccination. We identified risk factors for Q fever seropositivity on farm and animal level in order to update control measures and to provide targeted advice for the Dutch dairy goat sector.

## Methods

### Study design and sampling strategy

The study was designed as a cross-sectional study. In March 2009, 357 dairy goat farms with more than 100 dairy goats were present in the Netherlands and approached for participation. These farms are considered commercial farms and include the size of all known clinically infected goat farms with Q fever. The wide range in herd size allows studying the influence of this size on the infection risk. For all farms, results from bulk milk monitoring using PCR were available from October 2009 until October 2010. Farms with less than 100 goats and farms with a goat population completely vaccinated during the voluntary vaccination campaign in 2008 (approximately 36,000 goats) were excluded. To estimate farm prevalence, 110 farms should be included based on an expected prevalence of 50%, with 95% confidence, 10% accuracy and 90% sensitivity of the serological test used [21]. Based on an assumed within-herd prevalence of 16% [16,19] and a herd size varying between 100 and 4000 goats, 21 goats per farm were to be screened for *C. burnetii *infection. On participating farms, the private veterinary practitioner collected serological samples from the jugular vein of goats before the vaccination campaign in 2009 started. Written informed consent was obtained from each participating farm. As the investigation by the Animal Health Service of goat serum samples taken by the private veterinary practitioner could be considered as part of regular and routine clinical-diagnostic care, no official review and approval of the Animal Welfare Commission was needed.

Samples were collected in the mandatory vaccination area of 2009 between May and September 2009 (91% of samples were taken in May and June 2009) while samples outside this area were collected between July 2009 and May 2010 (81% of samples were taken from October 2009 until January 2010). A farm questionnaire was sent by e-mail or regular mail to all participating farms between October 2009 and May 2010 and completed by the farm owner or farm manager. The questionnaire addressed the general farm situation, number of lambs and goats, housing characteristics, vermin control and manure handling in 2008 (the year before the mandatory hygiene protocol was implemented), general health status and reproductive problems (including abortion rates) of the herd, breeding information, annual milk production and farm management, including biosecurity and hygiene measures for own staff and farm visitors.

### Laboratory analysis

Individual goat serum samples were tested with an ELISA test (Ruminant serum Q Fever ELISA kit, Laboratoire Service International, Lissieu, France) on *C. burnetii *specific antibodies with a single 1:400 serum dilution. All steps were carried out according to the instruction of the manufacturer. A goat was considered ELISA-positive if the optical density percent was 40 or higher, otherwise negative. A farm was considered positive if at least one goat on the farm was classified positive.

### Data analyses

#### Non-response analysis

Participating and non-participating farms were compared with respect to bulk milk PCR results (LSI TaqVet *Coxiella burnetii*, LSI, Lissieu, France), herd size, goat, sheep and cattle density, degree of urbanization and region where farms were located to study the representativeness of participating farms. Categorical variables among participating and non-participating farms were compared with a chi-square or Fisher exact test while numerical variables were compared using the Wilcoxon rank sum test.

#### Descriptive statistics and risk factor analysis

Animal and farm prevalence of *C. burnetii *with corresponding exact 95 percent confidence intervals were calculated. First, frequency tables of categorical variables were analysed and distributions of continuous variables were studied, and if not linearly related to the outcome variable divided into classes based on biological arguments, and if these were lacking based on medians. Potential risk factors on farm level were analysed by logistic regression (PROC LOGISTIC, SAS Institute Inc., 2004), and on animal level by generalized linear regression analysis accounting for farm effect (PROC GENMOD, SAS Institute Inc., 2004). For the latter, an exchangeable correlation covariance structure fitted best and was used to account for within-herd variation. First, univariable analyses were performed. In multivariable analysis, all variables with a p-value below 0.20 in the univariable analyses were included. For multivariable analysis on farm level, we excluded variables with less than 10% of data in one risk category. Proxy outcomes such as bulk milk status and mandatory vaccination area were not included in multivariable analyses. A backwards elimination procedure was performed until all variables were significant at 10% significance level in the likelihood ratio test. Two-way interactions between biological plausible and significant variables in the multivariate model were investigated. For the final model on farm level, model fit was assessed with the Hosmer-Lemeshow-Goodness-of-fit test (Hosmer and Lemeshow, 1989). Model fit on animal level was assessed by the QIC (Quasilikelihood under the Independence Model Criterion) goodness of fit statistic for GEE models.

## Results

### Non-response analysis

**Figure 1:**
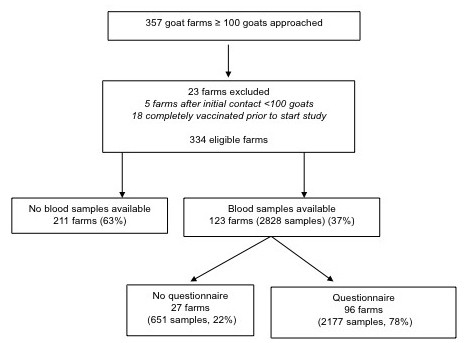
**Study participation of invited commercial dairy goat farms (> 100 goats), The Netherlands, 2009-2010**.

Of the 357 approached farms, 23 farms were excluded as they were not eligible for participation due to complete vaccination in 2008 or a herd size < 100 goats. In total, 123 dairy goat farms (36.8%) out of 334 eligible farms were willing to cooperate (Figure 1). Three additional dairy goat farms (0.9%) with only three goat sera were excluded from analysis and considered as a non-participant. Farms in the mandatory vaccination area participated more often than farms outside this area, 46.1% versus 30.1% (p < 0.05). The median number of goats among participating farms was 782 goats compared to 689 goats in the non-participating farms (p < 0.05). Bulk milk PCR-positivity did not differ between participating and non-participating farms, 24.4% and 22.8%, respectively. Participating and non-participating farms were also comparable with regard to location in rural areas (95.1% versus 97.2%), municipal cattle density (median: 121 versus 117), sheep density within 10 kilometres from farm (median: 21 versus 16) and affiliation to an organic goat farming cooperative (11.4% versus 13.4%).

### Descriptive results

From the 123 participating farms, 51 farms (41.5%) were located in the two southern provinces, 44 (35.8%) in the eastern part, 16 (13.0%) in the western part and 12 (9.7%) in the northern part of the country. The majority of farms (95.8%) were located in rural areas (< 500 addresses/km^2^). A farm questionnaire was available for 96 (78.0%) of the 123 dairy goat farms. Consequently, the investigation of risk factors was conducted in this subsample of 96 farms. Participating goat farms started between 1975 and 2009 (median: 1997, interquartile range (IQR) 1995-2000). The Dutch White goat was present on all farms. Additionally, other goat species, such as Toggenburger, Dutch pied original, Anglo-Nubian, alpine or mixed breeds were present at 50 farms (52.1%). The median annual milk production per goat was 1000 litres (IQR 900-1150 litres). Fourteen farms (14.6%) reported abortion percentages of 4% or higher in the period 2007-2009 (n = 11) and/or experienced an abortion wave due to Q fever since 2005 (n = 7).

### Seroprevalence

**Figure 2:**
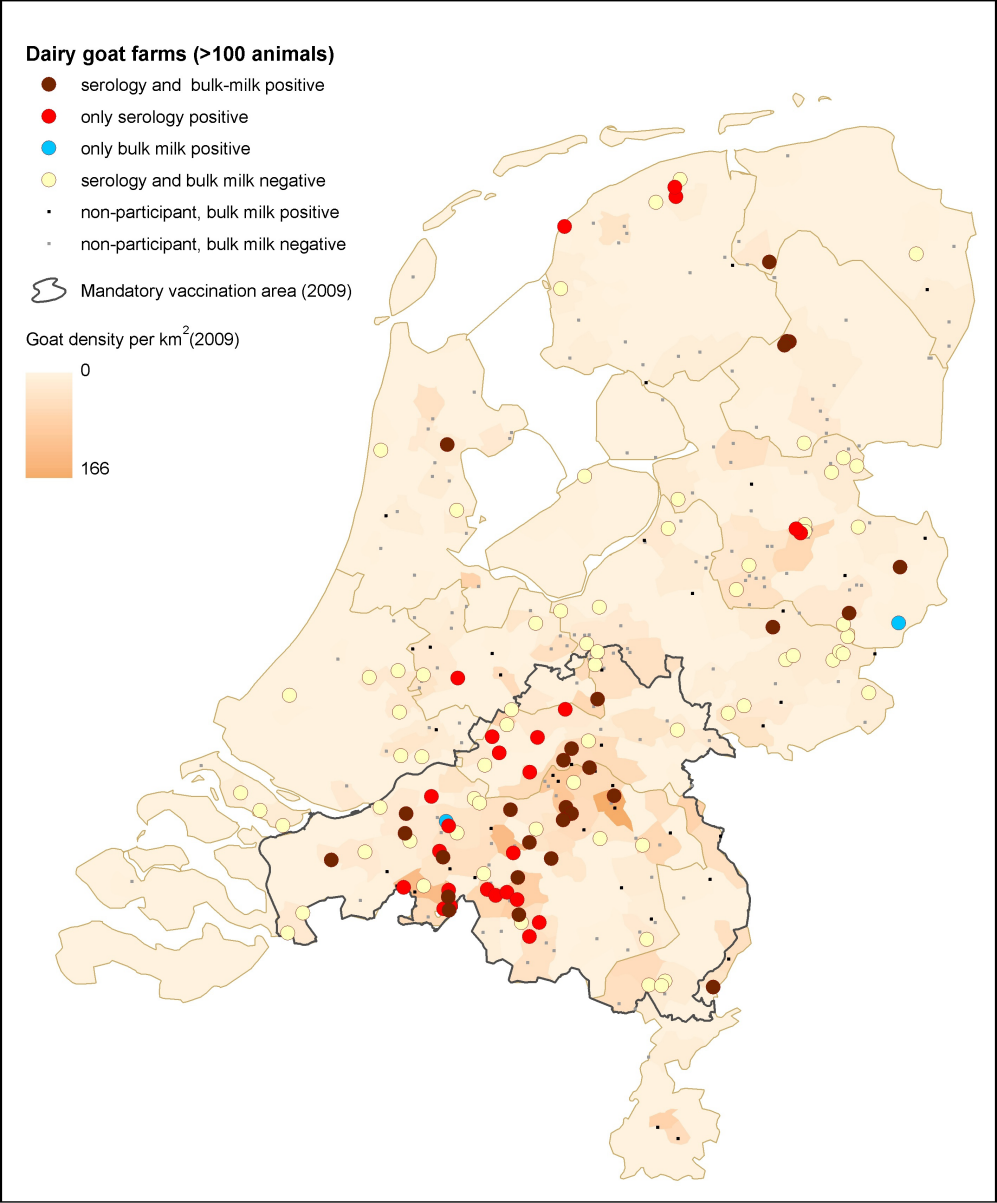
**Serological status of participating farms and bulk milk PCR status of eligible dairy goat farms**. Map of the Netherlands showing the 12 provinces, the mandatory vaccination area 2009 and the geographic locations of 123 participating dairy goat farms (median 782 goats, range 120-4146) and 211 non-participating farms (median 689 goats, range 105-4733), the serological and bulk milk PCR status of participating farms and bulk milk PCR status only of non-participating farms

A total of 2,828 serum samples were taken at the 123 participating farms. At 101 farms (82.1%), 21 samples per farm were taken as planned, while at the other 22 farms the median number of samples was 22 (range 13-116). Of these 2,828 samples, 21.4% were seropositive (exact 95% CI: 19.9-23.0) (Figure 2). At least one positive goat serum sample was found on 53 out of the 123 farms (43.1%; exact 95% CI: 34.2-52.3). On these 53 positive farms 46.6% of tested animals were seropositive (exact 95% CI: 43.9-49.3%). The prevalence of seropositive goats per farm varied between 4.8% and 95.2% (mean 46.0%, median 45.8% positive goats per farm, inter-quartile range 23.8%-63.6%). The average herd size on positive farms was 1,116 goats (median 890 goats, range 121-4,146) while the average herd size of negative farms was 793 goats (median 729 goats, range 120-2,970). Within the mandatory vaccination area, 58.5% of farms were classified positive compared to 25.9% of goat farms outside this area. Samples within the mandatory vaccination area were almost all taken during the end of the lambing season in 2009 (May-June) while 82.9% of samples outside this area were taken during the next lambing season in 2010 defined as December 2009 until May 2010. Median age, known for 1,474 goats, was 2.3 years. Seroprevalence increased with age: 5.8% for goats younger than 1 year, 15.7% between 1-3 years and 26.1% older than 3 years. Prevalence of the group that had lambed at least once was significantly higher than in the nulliparous group (19.4% (n = 1,251) vs. 11.9% (n = 236); for 1,341 goats lambing status was unknown.

### Comparison of bulk milk and serological status

From the 123 participating farms, 30 were bulk milk PCR positive (24.4%; exact 95% CI: 17.1-33.0). Among these 30 farms, 28 (93.3%) were also classified positive based on individual animal sera, with an average animal prevalence of 54.4% per positive farm. One farm was serologically negative in May 2009, but tested bulk milk-positive in November 2009, while the other farm was serologically negative in January 2010, and tested bulk milk-positive end March 2010. At 25 (26.9%) of the 93 bulk milk-negative farms at least one goat was positive, with an average animal prevalence of 36.5% per positive farm.

### Univariable analyses on farm level

Risk factors associated (p < 0.20) with farm seropositivity were herd size larger than 800 goats, location in mandatory vaccination area, farm location within 8 km from nearest bulk milk PCR-positive farm, high goat and cattle density, 3 or more stables, use of artificial insemination, having a dog at the farm, having a dog or a cat in the goat stable, unknown status of signs of vermin in roughage or litter, use of silage feed, maize and other feed such as lucerne or pulp, straw imported from abroad or unknown origin, use of a fodder mixer, controlling nuisance animals (e.g. wild birds) by covering air spaces, abortion percentage ≥ 4% during 2007-2009 or known history of abortion wave due to Q fever since 2005, spread of manure to other places than own farm or direct environment, two or more lambing seasons annually and having a closed or open tenure versus a semi-closed tenure (e.g. only bucks supplied). Protective factors were keeping rabbit(s) or pet birds, sidewall ventilation, and goat supply from the provinces of Friesland and Overijssel (Table 1).

**Table 1:** Univariable logistic regression of farm-based factors associated with serological Q fever infection on farm level

Variable	Category	N (%)	Prev(%)	OR	95% CI	P-value
Herd size (number of goats according to UBN registry)	≥ 800	47 (49.0)	59.6	3.7	1.6-8.6	0.0020
	< 800	49 (51.0)	28.6	Ref		
Mandatory vaccination area 2009	Inside	53 (55.2)	58.5	4.1	1.7-9.9	0.0010
	Outside	43 (44.8)	25.6	Ref		
Goat density per km^2 ^ within 5 km radius from farm	≥ 25	48 (50.0)	56.3	2.8	1.2-6.5	0.0130
	< 25	48 (50.0)	31.3	Ref		
Cattle density per km^2 ^ in farm municipality (excl. meat calves)	≥ 100	67 (69.8)	50.8	2.7	1.1-7.0	0.0329
	< 100	29 (30.2)	27.6	Ref		
Distance to nearest bulk milk PCR-positive farm (km)	0- < 4	29 (30.2)	72.4	10.0	3.4-29.1	< .0001
	4- < 8	19 (19.8)	57.9	5.2	1.7-16.5	
	≥ 8	48 (50.0)	20.8	Ref		
Number of stables	1-2 stables	40 (41.7)	35.0	Ref		0.1424
	≥ 3 stables	56 (58.3)	50.0	1.9	0.8-4.3	
Use of artificial insemination	Yes	26 (27.1)	61.5	2.7	1.1-6.7	0.0370
	No	70 (72.9)	37.7	Ref		
Having at least one dog at the farm	Yes	84 (87.5)	47.6	4.6	0.9-22.0	0.0337
	No	12 (12.5)	16.7	Ref		
Having at least one rabbit at the farm	Yes	26 (27.1)	30.8	0.5	0.2-1.2	0.1138
	No	70 (72.9)	48.6	Ref		
Having at least one pet bird at the farm	Yes	25 (25.0)	28.0	0.4	0.2-1.1	0.0606
	No	71 (75.0)	49.3	Ref		
Dog(s) in goat stable	Yes	60 (62.5)	51.7	2.4	1.0-5.8	0.0415
	No/unknown	36 (37.5)	30.6	Ref		
Cat(s) in goat stable	Yes	34 (35.4)	58.8	2.6	1.1-6.1	0.0275
	No/unknown	62 (64.6)	35.5	Ref		
Signs of vermin (mice, rats, birds) in roughage or litter during past 12 months	Unknown	14 (14.6)	64.3	2.7	0.8-8.7	0.0944
	Known (yes or no)	82 (85.4)	40.2	Ref		
Feeding silage	Yes	64 (66.7)	51.6	2.7	1.1-6.8	0.0269
	No	32 (33.3)	28.1	Ref		
Feeding maize	Yes	40 (41.7)	55.0	2.2	1.0-5.0	0.0602
	No	56 (58.3)	35.7	Ref		
Use of lucerne, pulp feed or other roughage/litter	Yes	24 (25.0)	58.3	2.2	0.9-5.6	0.0972
	No	72 (75.0)	38.9	Ref		
Origin of straw	Abroad/unknown	59 (61.5)	49.2	1.8	0.8-4.2	0.1757
	No straw or domestic straw	37 (38.5)	35.1	Ref		
Feeding method	Hand/wheelbarrow	31 (32.3)	22.6	Ref		0.0202
	Fodder mixer	56 (58.3)	57.1	4.6	1.7-12.4	
	Automatic	9 (9.4)	33.3	1.7	0.3-8.7	
Sidewall ventilation	Yes	38 (39.6)	34.2	0.5	0.2-1.2	0.1252
	No	58 (60.4)	50.0	Ref		
Control nuisance animals (e.g. wild birds) in 2008	Yes, by covering air spaces	15 (15.6)	73.3	2.1	1.3-15.4	0.0409
	Yes, only via another ways (a.o. capture cage)	13 (13.5)	38.5	1.0	0.3-3.4	
	Not applicable	68 (70.8)	38.2	Ref		
Percentage of aborting goats or goats with stillbirth in 2007-2009 or known history of abortion wave due to Q fever since 2005	< 4%	82 (85.4)	39.0	Ref		0.0234
	≥ 4% and/or abortion wave	14 (14.6)	71.4	3.9	1.1-13.5	
Spread of manure	On or near own farmland	79 (82.3)	39.2	Ref		0.0497
	To other places	15 (15.6)	66.7	3.1	1.0-9.9	
Lambing periods in 2009	≤ 1	57 (59.4)	31.6	Ref		0.0035
	≥ 2	39 (40.6)	61.5	3.5	1.5-8.1	
Type of tenure	Completely closed	27 (28.1)	55.6	2.4	0.9-6.1	0.1158
	Closed for female goats only	52 (54.2)	34.6	Ref		
	No, not closed	16 (16.7)	56.3	2.4	0.8-7.6	
Goat supply from the province of Friesland	Yes	19 (19.6)	26.3	0.4	0.1-1.2	0.0806
	No	77 (80.2)	48.1	Ref		
Goat supply from the province of Overijssel	Yes	10 (10.4)	20.0	0.3	0.1-1.4	0.0961
	No	86 (89.6)	46.5	Ref		

Frequency (N), prevalence (Prev), odds ratio (OR), and 95% confidence interval (95%CI) of variables with Likelihood ratio test P values < 0.20 (96 farms, 43.1% positive farms based on total sample of 123 farms and 2,828 goats)

### Multivariable analysis on farm level

A total of 21 variables were included in the initial multivariable model. Artificial insemination strongly correlated with large herd size and was excluded from the model. Supply from the provinces of Friesland and Overijssel provinces was not included in the model as possible protective factor because overall goat supply or supply from other provinces were not significant risk factors in the univariable analysis. Having a rabbit or a pet bird was also not included as it correlated inversely with having a dog. The following nine variables remained independently associated with seropositivity in the final model (Table 2): farm located within 8 km proximity to a bulk milk PCR-positive dairy goat farm, high cattle density, controlling nuisance animals by covering air spaces, presence of cats and/or dogs in the goat stable, straw imported from abroad or unknown origin, a herd size of 800 goats or more and unknown status of signs of vermin in roughage or litter. Interaction terms were not statisticially significant and did not improve the model. The Hosmer and Lemeshow Goodness-of-Fit test showed no lack of fit of the model (P = 0.60).

**Table 2:** Multivariable logistic regression of farm-based factors associated with serological Q fever infection on farm level

Variable	Category	N (%)		Prev (%)	aOR	95% CI
Distance to the nearest bulk milk PCR-positive farm (km)	< 8	48	50.0	66.7	12.9	3.0-54.8
	≥ 8	48	50.0	20.8	Ref	
Cattle density per km^2 ^in farm municipality (excl. meat calves)	≥ 100	67	69.8	50.8	14.4	2.7-78.4
	< 100	29	30.2	27.6	Ref	
Herd size (number of goats according to UBN registry)	≥ 800	47	49.0	59.6	2.8	0.8-9.4
	< 800	49	51.0	28.6	Ref	
Control nuisance animals (e.g. wild birds) in 2008	Yes, by covering air spaces	15	84.4	73.3	48.8	4.0-591.2
	By other ways or not applicable	81	15.6	38.3	Ref	
Dogs(s) in goat stable	Yes	60	62.5	51.7	3.8	1.0-14.2
	No/unknown	36	37.5	30.6	Ref	
Cat(s) in goat stable	Yes	34	35.4	58.8	6.3	1.5-25.8
	No/unknown	62	64.6	35.5	Ref	
Origin of Straw	Abroad/unknown	59	61.5	49.2	5.0	1.3-19.6
	No straw or domestic straw	37	38.5	35.1	Ref	
Signs of vermin (mice, rats, birds) in roughage or litter during past 12 months	Unknown	14	14.6	64.3	4.3	0.8-22.3
	Known (yes or no)	82	85.4	40.2	Ref	

Factors associated with Q fever on farm level with their frequency (N), prevalence (Prev), adjusted odds ratio (aOR) with corresponding 95% confidence interval (95% CI) in the final multivariable logistic model (96 farms; 43.1% positive farms based on total sample of 123 farms and 2,828 goats)

### Univariable risk factor analyses on animal level

The same variables as in the analysis on farm level were identified on animal level, except for having a dog in the stable and straw imported from abroad or unknown origin. Additionally the following univariable risk factors were identified on animal level: mechanic ventilation in the stable, use of windbreak curtain only or in combination with wind shields, presence of few nuisance animals (e.g. wild birds), while additional univariable protective factors on animal level were presence of the Anglo-Nubian goat and keeping laying hens (Table 3).

**Table 3:** Univariable logistic regression of farm-based factors associated with serological Q fever infection on animal level

Variable	Category	N	Prev. (%)	OR	95% CI	P-value
Herd size (according to UBN registry)	< 800	1164	13.8	Ref		0.0017
	≥ 800	1013	30.2	3.3	1.6-7.0	
Mandatory vaccination area	Inside	1116	28.2	3.3	1.5-7.3	0.0042
	Outside	1061	14.3	Ref		
Goat density per km^2 ^in 5 km radius from the farm	< 25	1167	18.2	Ref		0.0805
	≥ 25	1010	25.3	1.9	0.9-3.9	
Cattle density per km^2 ^in farm municipality (excl. meat calves)	< 100	643	11.2	Ref		0.0084
	≥ 100	1534	25.8	3.5	1.4-8.9	
Distance to nearest bulk milk PCR-positive farm (km)	0- < 4	688	37.8	5.0	2.2-11.6	< .0001
	4- < 8	400	25.3	3.2	1.1-8.9	
	≥ 8	1089	9.7	Ref		
Anglo-Nubian goat	Present	402	11.9	0.5	0.2-1.1	0.0694
	Absent	1775	23.6	Ref		
Number of stables	1-2 stables	983	14.2	Ref		0.0046
	≥ 3 stables	1194	27.4	3.0	1.4-6.5	
Use of artificial insemination	Yes	572	34.6	2.7	1.3-5.6	0.0181
	No	1585	17.0	Ref		
Presence of laying hens at the farm	Yes	359	8.9	0.3	0.1-0.9	0.0184
	No	1818	23.9	Ref		
Having at least one dog at the farm	Yes	1927	23.5	Ref		0.0530
	No	250	5.6	0.2	0.0-1.0	
Having at least one rabbit at the farm	Yes	608	6.4	0.2	0.1-0.6	0.0008
	No	1569	27.3	Ref		
Having at least one pet bird at the farm	Yes	501	9.4	0.3	0.1-0.9	0.0132
	No	1676	25.1	Ref		
Cat(s) in goat stable	Yes	786	24.2	1.7	0.8-3.4	0.1718
	No/unknown	1391	19.9	Ref		
Signs of vermin (mice, rats, birds) in roughage or litter during past 12 months	Unknown	364	41.8	2.8	1.2-6.2	0.0471
	Yes or No	1813	17.4	Ref		
Feeding silage	Yes	1498	26.1	Ref		0.0218
	No	679	11.2	0.4	0.2-0.9	
Feeding maize	Yes	872	28.8	Ref		0.0333
	No	1305	16.6	0.5	0.2-0.9	
Use of lucerne, pulp feed or other roughage/litter	Yes	505	28.1	1.8	0.9-3.7	0.1445
	No	1672	19.4	Ref		
Feeding method	With hand/wheelbarrow	772	13.5	0.3	0.1-0.9	0.0299
	With fodder mixer	1191	27.0	Ref		
	Automatic	214	19.6	0.5	0.1-1.9	
Type of ventilation system of stables	Mechanic ventilation	593	30.5	2.0	1.0-4.3	0.0899
	No mechanic ventilation	1584	18.1	Ref		
Use of windbreak curtain and/or windshields	Windbreak curtain	799	30.5	3.7	1.5-9.3	0.0198
	Only wind shields	937	18.8	1.6	0.6-4.3	
	None	441	10.7	Ref		
Presence of nuisance animals (e.g. wild birds) in the stable	Yes, many	388	12.6	0.6	0.2-2.0	0.1206
	Yes, few	880	27.3	1.7	0.8-3.7	
	No	889	20.0	Ref		
Combat of nuisance animals (e.g. wild birds) in 2008	Via covering air spaces	349	38.7	3.0	1.4-6.3	0.0721
	Yes, only via other ways (e.g. capture cage)	274	21.2	1.3	0.4-4.3	
	Not applicable	1554	17.6	Ref		
Spread of manure	To other places/not applicable	352	30.7	2.1	1.0-4.7	0.1076
	On farmland or near environment	1825	19.7	Ref		
Lambing periods in 2009	≤ 1	1351	15.6	Ref		0.0039
	> 1	826	31.0	3.0	1.5-6.1	
Percentage of aborting goats or goats with stillbirth in 2007-2009 or known history of abortion wave due to Q fever since 2005	< 4%	1882	18.4	Ref		0.0251
	≥ 4%	295	40.7	3.4	1.5-7.6	
Type of Tenure	Completely closed	590	29.5	2.3	1.0-5.0	0.0898
	Only closed for female goats	1217	16.1	Ref		
	Not closed	349	27.8	2.2	0.8-5.6	
Provinces where supplied animals originated	Provinces of Friesland and/or Overijssel	639	8.0	0.3	0.1-0.7	0.0028
	Supply from other provinces or no supply	1538	27.1	Ref		

Factors associated with Q fever with their frequency (N), animal prevalence (Prev), odds ratio (OR), and 95% confidence intervals (95% CI) with P value of the univariable generalized linear regression < 0.20 on animal level accounting for random herd effect (2,177 animals from 96 farms; 21.4% seropositive animals and 43.1% positive farms based on total sample of 123 farms and 2,828 goats)

### Multivariable risk factors analysis on animal level

The variable presence of a dog on the farm was not included on animal level, as the reference category was too small as almost all farms (88%) had a dog. Seven variables remained in the multivariable model on animal level of which five also were present in the final model on farm level i.e. farm located within 8 km proximity from a bulk milk PCR-positive dairy goat farm, high cattle density, presence of a cat in the goat stable, controlling nuisance animals by covering air spaces and unknown status of vermin in roughage or litter. Additional risk factors on animal level were use of artificial insemination and use of windbreak curtain only or in combination with wind shields compared to none of these (Table 4). Within-farm variation accounted for 34.6% of all non-explained variance of the model.

**Table 4:** Multivariable logistic regression of farm-based factors associated with serological Q fever infection on animal level

Variable	Category	N (%)		Prev (%)	aOR	95% CI
Distance to the nearest bulk milk PCR-positive farm (km)	< 8 km	1088	50.0	33.2	3.2	1.4-7.3
	≥ 8 km	1089	50.0	9.73	Ref	
Cattle density per km^2 ^in farm municipality (excl meat calves)	< 100	643	29.5	11.2	Ref	
	≥ 100	1534	70.5	25.8	4.5	2.0-9.9
Combat of nuisance animals (e.g. wild birds) in 2008	Yes, by covering air spaces	349	16.0	38.7	3.7	1.8-7.9
	By other ways or not applicable	1828	84.0	18.2	Ref	
Signs of vermin (mice, rats, birds) in roughage or litter during past 12 months	Unknown	364	16.7	41.8	3.3	1.4-7.9
	Known (yes or no)	1813	83.3	17.4	Ref	
Cat(s) in goat stable	Yes	786	36.1	24.2	2.6	1.2-5.6
	No/unknown	1391	63.9	19.9	Ref	
Use of windbreak curtain and/or windshields	Windbreak curtain	799	36.7	30.5	2.8	1.2-6.7
	Only wind shields	937	43.0	18.8	1.7	0.7-4.1
	None	441	4.9	10.6	Ref	
Artificial insemination	Yes	572	26.3	34.6	2.3	1.2-4.7
	No	1585	72.8	17.0		

Factors associated with Q fever with their frequency (N), animal prevalence (Prev), adjusted odds ratio (aOR) with corresponding 95% confidence interval (95% CI) in the final multivariable model on animal level with random herd effect (2,177 animals from 96 farms; 21.4% positive animals; 43.1% positive farms based on total sample of 123 farms and 2,828 goats)

**within-farm variation accounts for 34.6% of non-explained variance

## Discussion

The overall animal and farm seroprevalence of *C. burnetii *in dairy goats farms with ≥ 100 dairy goats observed in this study was 21.4% and 43.1% respectively. These seroprevalence estimates increased compared to the seroprevalence measured in 2008, when 14.7% of individual dairy goats were serologically positive and 17.9% of farms tested positive. The within-herd prevalence on positive dairy goat farms in our study was 46.6% compared to 32.1% (95%CI 28.4%-35.9%) in 2008 (van den Brom R, Moll L, Vellema P: Q fever seroprevalence in sheep and goats in the Netherlands in 2008, submitted). This study demonstrates substantial transmission of *C. burnetii *within and between dairy goat farms in recent years prior to the mandatory vaccination campaign in the Netherlands.

The relatively low overall participation rate of 37% probably reflects the reluctance to take part in the study at the same time as control measures increased, including finally the culling of pregnant goats at bulk milk PCR-positive farms. The overrepresentation of farms located in the mandatory vaccination area probably reflects that the risk perception of the farmers played a role. Because of the higher participation rate in this area, we might have overestimated the overall seroprevalence in eligible dairy goat farms in the Netherlands. As expected, dairy goat farms located in the mandatory vaccination area were more often seropositive in our study, as was previously observed in 2008 (van den Brom R, Moll L, Vellema P: Q fever seroprevalence in sheep and goats in the Netherlands in 2008, submitted). In contrast, the estimated seroprevalence might have been underestimated as the non-eligible farms probably overrepresented positive farms, such as farms with a clinical history of Q fever. These were prioritized for vaccination early 2009, and probably positive and suspected farms relatively more often volunteered for vaccination in the 2008 campaign. Nevertheless, the net effect of these biases are thought to be limited, as bulk milk-positive farms were equally represented among participating and non-participating farms. As the diversity in farms, also outside the vaccination area, was still large, effect on the risk factor analyses is considered limited and results are considered generalizable to all commercial dairy goat farms in the Netherlands.

Small ruminant studies have shown that goats test significantly more often serologically positive during pregnancy and in the periparturient period compared to early pregnancy or non-pregnant period (van den Brom R, Moll L, Vellema P: Q fever seroprevalance in sheep and goats in the Netherlands in 2008, submitted), [21]. Different sampling periods in our study, mainly at the end of the lambing season in 2009 inside the vaccination area and in the beginning of the lambing season 2010 outside the vaccination area, make it difficult to disentangle the possible effect of seasonal sampling on the observed significant regional differences. We think this study shows a true higher seroprevalence in the mandatory vaccination area as it (1) confirms the significant difference already observed in 2008 in the south-eastern part of the country and (2) reflects the major burden of human and veterinary clinical Q fever cases that occurred in the south-eastern part of the country [1,2,10]. A distinction with ELISA between IgG phase 1 and 2 antibodies might have helped to distinguish more recent infection and older infections in animals to assess such a sampling effect [22].

Seroprevalence of *C. burnetii *in goats has been studied in several countries [16]. Comparison should be done with caution as study populations and study years vary and different serological assays with different performance are used [23,24]. The goat seroprevalence was 8.8% in Albania [25] and 6.5% in Northern Greece [26]. In Spain, the goat and farm prevalence was 8.7% and 45%, respectively, based on ELISA [19] similar to results of a smaller study from Northern Ireland (goat seroprevalence 9.3%, farm prevalence 42.9%) [17], and in Sardinia in 1999-2002: goat prevalence 13% and farm prevalence 47% using an alternative criterion of two or more seropositive animals per farm [18]. In our study, the farm seroprevalence was 38.2% (95%CI 29.6%-46.8%) using the same criterion, so only slightly lower than with at least one positive goat as criterion for a positive farm. Clearly different goat prevalences were observed in Poland, with the absence of *C. burnetii *IgG phase 2 antibodies in 918 goats from 48 herds [27] while high estimates were observed in Cyprus (48.2% in 420 random goats) [28] and in Gran Canaria island, Spain (60.4% in 733 goats) [29]. Ignoring these last exceptions, the overall goat prevalence of 21.4% observed in our study was relatively high compared to other European seroprevalence studies (6.5%-13%), while the farm prevalence falls within the range of farm prevalences (43-47%) in other European countries. The within-herd prevalence of 46.6% among the positive farms indicates strong circulation of the bacterium within the herds, suggesting farm conditions or practices favoring spread, such as a relatively large number of goats per farm, year-round housing in deep litter stables or reflects circulation of a unique efficiently spreading strain. In France, goat herds with a within-herd prevalence over 40% had the highest proportion of shedder goats and highest averages of shedding quantities as determined by real-time qPCR on vaginal swabs, representing a high risk level for environmental contamination and by that transmission within farms [20]. At about one quarter of the bulk milk PCR-negative farms, on average 37% of the goats tested seropositive. This might be explained by the fact that not all seropositive goats shed the bacterium in milk and that excretion of the bacterium is intermittently [7,15]. Besides, antibodies persist in goats [9], finding still positive serology but no actual excretion of DNA which is measured in the bulk milk monitoring program.

Considering the risk factors analyses, the exposure information collected in the farm questionnaire is not necessarily related to the relevant time period for seroconversion as we do not know at what moment the actual infection with *C. burnetii *occurred in serologically positive goats. However, since the median age of tested goats was 2.3 years, and infections especially occur during the first pregnancy of nulliparous goats (between 1-2 years of age) it is plausible that infection in the majority of goats occurred during the periods covered in the questionnaire. It is most likely that goats on these farms get infected the same way as humans, i.e. by inhalation of *C. burnetii *infected aerosols [4], as indicated by the increased risk of a farm location within 8 km of a bulk milk-positive small ruminant farm. From literature we know that herd size and high farm and animal densities can augment the risk for acquisition of (respiratory) zoonoses, for example in swine diseases and avian influenza in poultry [30,31]. In our study, we found that farms with more than 800 goats had a higher risk to be positive than smaller farms. This corresponds with Rupanner *et al. *who observed in the 1970s an increased infection risk of goats with *C. burnetii *with increasing herd size [32]. Similar associations with herd size were found for Q fever in dairy cattle [17,33]. This can be explained by a larger population at risk, an increased risk of introduction and transmission of pathogens within and between herds for instance by larger amounts of feed, animal supply and more professionals working at or visiting the farm. In addition, farm management practices or environmental characteristics related to large farms but not covered in our questionnaire might play a role in the observed increased risk. As about 35% of the unexplained variance in the model was explained by the farm-effect, relevant underlying factors might have been missed. Therefore, an advice to limit the herd size without further changes in farm management does not necessarily guarantee a reduction in infection risk. Artificial insemination was an independent risk factor at animal level and found to be related to farms with a herd size over 800 goats. Artificial insemination can therefore be an indirect marker of farm management practices in larger farms that were not covered in the questionnaire. From cattle studies, it is known that viable C. *burnetii *is detected in semen of seropositive bulls indicating the possibility of sexual transmission [34]. Between 3000 and 4000 inseminations each year are carried out by the main goat artificial insemination (AI) cooperative using fresh semen from the Netherlands and frozen semen from French or Dutch origin. Since end of 2008, AI bucks are routinely screened for presence of *C. burnetii*. In a targeted survey, so far, goat semen samples from 300 bucks present on bulk milk-positive farms were all negative (personal communication, P. Vellema, Animal Health Service). High cattle density in the municipality where the farm was located was also an independent risk factor, indicating the presence of one or several cattle farms in the same municipality as the goat farm. A recent review on *C. burnetii *infection in domestic ruminants suggested a higher seroprevalence in cattle compared to goats and sheep [16]. In the Netherlands, a prevalence of bacterial DNA of 56.6% in cattle bulk tank milk was found as compared to 24.4% bacterial DNA in goat bulk tank milk among participating farms in our study, confirming widespread circulation of the bacterium among cattle [35]. However, an association with cattle density was not observed when the outcome variable 'bulk milk PCR-positivity' was used instead of ELISA-seropositivity (data not shown). Therefore, it is hypothesized that cattle especially played a role in the more historical infections in goats, while spread between dairy goat herds is responsible for the more recent infections and a large part of the epidemic observed since 2007. The serological status of cattle and foremost comparison of *C. burnetii *isolates by subtyping in different ruminant species might help to elucidate the transmission pathways between different species of ruminants and to humans. So far, one unique genotype predominated in dairy goats herds, although at 50% of the farms at least one additional genotype was observed [36]. Very sparse data on cattle isolates in the Netherlands suggest different subtypes from those found in goats, sheep and humans [37]. More and nationwide representative data are urgently needed to confirm these distinct types for cattle, and to study if some cattle types match with the non-dominant genotypes regularly observed at dairy goat farms.

Previous ruminant studies have shown that farm management practices can influence the seroprevalence of *C. burnetii *[17,38]. Straw, used widely as bedding in deep litter stables, could be a way in which Q fever was introduced in the Dutch dairy goat farms as import of straw from abroad or unknown origin was an independent risk factor. Farmers indicated straw was most often imported from Germany and France, which are endemic countries for Q fever. Microbiological examination from straw originating from France showed presence of *C. burnetii *by PCR, although the method of sampling does not exclude contamination at the farm [39]. Contact with straw and other farm products was also a risk factor for humans in the first documented outbreak in 2007 and in international outbreak studies [40-42]. The presence of dogs and cats in the goat stable was related to a seropositive Q fever status of dairy goat farms. Furthermore, the seven farms without companion animals were all seronegative. This suggests introduction of *C. burnetii *or facilitation of within farm-spread by infected companion animals. In a study in Cyprus, risk factors for Q fever abortions compared to abortions of other causes were studied in a convenience sample of ruminant farms including only two goat farms; among others presence of dogs and cats were on farm risk factors [38]. Pets, especially during kidding, have been associated with outbreaks in the past [5,6]. In a Dutch study in the early 1990s, 13.2% of dogs and 10.4% of cats tested positive for *C. burnetii *by ELISA [43]. To study the role of companion animals in current transmission, an update of this study, ideally also looking at shedding by PCR, is needed.

Covering airspaces in the stable to control nuisance animals, such as wild birds, unexpectedly was an independent risk factor. As wild birds may play a role in the transmission within and between farms and were the cause of a familial Q fever outbreak [44] a protective effect was expected if any. However, presence of wild birds in the stable was not a risk factor in the multivariable analysis but mainly indicated the farmer actively controlled nuisance animals such as by covering airspaces. In addition, we found at animal level an increased risk for farms that use windbreak curtain, sometimes in combination with windshields. These two risk factors could point at a more air-locked stable, facilitating accumulation of *C. burnetii *inside the stable, which may promote spread within the herd. This accumulation risk was indirectly shown in the study from Cyprus where a high frequency of litter cleaning was found to be a protective factor [38]. Although a less confined farm might limit the within-herd spread, such open constructions can be a risk for aerosol spread to other farms and persons in the near environment. Presence of mice and rats in the stable was not found to be a risk factor in our study, although a recent study showed presence of *C. burnetii *in rats at livestock farms in the Netherlands [45]. Whether vermin are able to maintain the transmission cycle and are able to (re)introduce Q fever at farms is currently under investigation.

## Conclusions

This study shows that before the start of mandatory vaccination of small ruminants in 2009-2010, the seroprevalence of *C. burnetii *antibodies in goats at commercial dairy goat farms has increased compared to a study carried out in 2008 in the Netherlands. The overall goat prevalence of 21.4% was considerably high, but the farm prevalence of 43.1% was comparable to generally observed seroprevalences in other European countries. On positive dairy goat farms, the within-herd prevalence was 46.6%, reflecting high circulation of *C. burnetii *within a farm and a risk for environmental contamination and spread. In general, the risk for farms and dairy goats to acquire a *C. burnetii *infection seems to be multifactorial. The two strongest associated risk factors, proximity of bulk milk-positive small ruminant farms and a high cattle density, suggest aerosol spread as an important route of infection of the dairy goat farms. Furthermore, other risk factors identified possible vehicles for introduction, spreading and/or persistence within farms, such as import of straw from abroad, access to the goat stable of cats, dogs and use of artificial insemination, and covering airspaces of the stable. Besides, larger farms with 800 or more goats seem to have an increased risk for infection, although it can not be concluded that this is entirely due to the size itself by the larger population at risk, combined with a general increased chance of introduction of pathogens in larger farms or is due to unmeasured farm characteristics strongly related to a large herd size. Based on our results, it is recommended to further prove the role in the current transmission of bedding material, goat semen and excreta from companion animals by microbiological testing. Simultaneously, as a precautionary measure general biosecurity measures should be taken next to advice on farm stable constructions targeted at avoiding access of companion animals and how to control nuisance animals in the goat stables to prevent introduction and minimizing airborne transmission from affected farms to prevent spread to humans and other farms.

## Competing interests

The authors declare that they have no competing interests.

## Authors' contributions

The study idea was conceived by BS and YD together with the Q-VIVE research group. BS, YD and PV participated in the design of the study. JH and YD participated in the acquisition of the data and coordinated logistics. PV provided previously acquired reference data. BS, SL, EG and YD carried out the statistical analysis. Data interpretation was done by all authors. BS and SL drafted the manuscript. All authors contributed to the critical revision of the manuscript for important intellectual content and have seen and approved the final draft.
